# Gender disparities in securing national clinical research program (PHRC-N) funding in France: a retrospective analysis of the 2022 campaign

**DOI:** 10.1186/s12961-026-01450-z

**Published:** 2026-03-08

**Authors:** Solène Mathieu, Constant Vinatier, Florian Naudet, Jean-Sébastien Allain

**Affiliations:** 1 Groupe Hospitalier Bretagne Sud, Lorient, France; 2 Rennes University, Rennes, France; 3 CHU Rennes, Inserm, EHESP, Institut de Recherche en santé, environnement et travail (Irset), UMR S 1085, Centre d’Investigation Clinique de Rennes (CIC 1414), Rennes University, Rennes, France; 4 Institut Universitaire de France (IUF), Paris, France

**Keywords:** Gender equity, Funding, Clinical research protocol, France

## Abstract

**Background:**

Despite progress in gender parity in medicine, women still face challenges in attaining senior academic and clinical roles. This study examines potential gender bias in awarding academic funding from the French National Clinical Research Program (PHRC-N), one of the main funders for clinical research in France.

**Methods:**

This retrospective observational study analysed 2022 PHRC-N campaign applications. Anonymized data were provided by the French ministry of health, including investigator demographics (gender, academic rank, location, prior funding) and project characteristics (discipline, number of centre, budget, sample size). Outcomes examined included funding success rates from initial application to award, amounts requested and amounts awarded. Statistical analyses followed a protocol registered prospectively with univariate and multivariate logistic regression.

**Results:**

Out of 321 eligible applications, 83/321 (26%) were led by women. Among these, 102/321 (32%) received funding, amounting to a total of 75 046 900 euros (i.e., 77 375 117 USD), with no significant difference observed between male (77/238, 32%) and female investigators (25/83, 34%; *p* = 0.707). Pre-selection rates and funding amounts awarded (781 243 ± 375 562 and 715 377 ± 251 307 euros for male and female investigators, respectively) also indicated no gender disparity. These findings remained consistent in multivariate analyses, which revealed higher success rates for projects led from the Île-de-France region (OR 1.79 [1.07–3.01], *p* = 0.027) and large-scale studies involving over 1000 participants (OR 3.83 [1.32–11.87], *p* < 0.016).

**Conclusions:**

There was an underrepresentation of women as principal investigators at the submission stage. However, no evidence of gender bias was found in PHRC funding outcomes for 2022. The lack of evidence for gender bias in funding outcomes should be interpreted with caution due to limited statistical power. Future research should examine long-term trends and broader funding programs to evaluate potential disparities in research opportunities and outcomes.

**Supplementary Information:**

The online version contains supplementary material available at 10.1186/s12961-026-01450-z.

## Introduction

Despite the increasing feminization of medical professions in France, gender disparities persist in achieving high-ranking academic positions, such as full professors (PU-PH) and associate professors (MCU-PH) [1–6]. Scientific publications are crucial for career advancement, and men dominate research output [7, 8]. Studies from other countries highlight gender disparities in access to research funding [9–13]. It is essential to identify and rectify funding allocation biases to promote gender equity in academia and enhance the research culture.

In France, the French National Clinical Research Program (Programme Hospitalier de Recherche Clinique, PHRC) is one of the primary academic funding mechanisms for medical research in France. Established in 1992, the PRHC is administered by the Directorate General for Healthcare Services (Direction Générale de l’Offre de Soins, DGOS) under the aegis of the Ministry of Health. Between 1993 and 2019, the PHRC has funded 3486 studies with a total investment to the tune of €1 billion (i.e. 1 031 023 497 USD) [14]. The PHRC is divided into several calls for proposals: PHRC-N (National), PHRC-I (Interregional) and PHRC-K (Cancer). PHRC-N focuses on national-level health projects excluding oncology, human immunodeficiency virus (HIV), hepatitis B virus (HBV), hepatitis C virus (HCV), severe acute respiratory syndrome coronavirus 2 (SARS-CoV-2) and emerging infectious diseases. PRHC-I is a regional version of PHRC-N. PHRC-K finances projects exclusively related to oncology. The funding process involves a two-stage selection with a preselection based on a letter of intent followed by the evaluation of a full proposal. Initially, the principal investigator drafts a letter of intent and submits it via a dedicated online platform. An independent jury composed of experts is assembled and conducts an initial evaluation to identify, and assess the relevance, safety and efficiency of the project. Principal investigators whose letters of intent are preselected may then submit their complete application (including the proposed study timeline, dated, signed and stamped protocol, budget grid, resumé of the coordinating investigator, resumé of the methodologist, commitment letters from centres and commitment letters from co-funders) [15]. This full application will again be reviewed by the same independent jury, which will select awardees [16].

Our research investigates possible gender disparities in obtaining PHRC-N funding during both phases of the process.

## Materials and methods

This retrospective observational study analysed the PHRC-N 2022 campaign (funding commitments in 2023). The study protocol was registered prospectively on the Open Science Framework: 10.17605/OSF.IO/DPFVJ on June 5, 2024.

### Data sources

The dataset included projects submitted to PHRC-N in 2022 (starting with a letter of intent). Excluded were studies without participant inclusion (i.e. retrospective studies on routinely collected data and meta-analyses). The amount obtained for funded research was retrieved from the Ministry of Health website: https://sante.gouv.fr/systeme-de-sante/innovation-et-recherche/l-innovation-et-la-recherche-clinique/appels-a-projets/article/les-projets-retenus.

The other data, including the author’s characteristics and project characteristics were obtained through the French Ministry of Health (DGOS) on 28 May 2024. Extracted variables included investigator gender, academic rank (full professor, associate professor, other), geographic location (Paris / Île-de-France, province), prior academic funding, resubmission status, number of centres, estimated sample size, project budget (in euros), awarded funding (in euros) and project discipline (medicine, surgery). These data contain sensitive information and have been anonymized.

### Explanatory variables and outcomes of interest

The primary outcome was the funding success rate from the letter of intent to final PHRC-N funding in 2022. Secondary outcomes included preselection success rates, funding success rates post-preselection and comparison of requested versus awarded funding amounts.

### Covariates

We adjusted for several covariates that could influence funding success. The covariates related to the principal investigator characteristics were medical specialty (medicine/surgery), geographic region (île-de-France / provinces), prior funding by the PHRC (yes/no), academic rank (full professor/associate professor/other/unknown). In addition, the project-related variables included the number of centres, sample size, project budget and whether the application was a resubmission of a previous project (yes/no).

### Statistical analysis

Quantitative data were described using means ± standard deviations (or medians and ranges when needed). Categorical data were presented as frequencies and percentages. Statistical comparisons used the Student’s *t*-test or Wilcoxon rank-sum test (when needed) for quantitative data and chi-square or Fisher’s exact test (when needed) for categorical data. Logistic regression analyses were conducted to determine the factors related to funding success. These included univariate and multivariate models that considered potential confounding variables described in the covariate section. A 5% significance threshold was applied, and all analyses were conducted in R (version 4.2.2). Analyses were performed between the 5 July and 30 July. The statistical code is openly accessible (10.17605/OSF.IO/3CR67).

### Confidentiality and ethics declaration

Anonymized data were provided by the DGOS to ensure confidentiality. Intellectual property elements, such as rationale and outcomes, were not accessible.

Ethics declaration: not applicable. This is a retrospective study using a database provided by the French Ministry of Health (DGOS). The data was provided in a fully anonymized form, making it impossible to re-identify individuals. In this context, no GDPR consent or ethical advice is required in France.

### Changes to the initial protocol

In the initial database, four observations had missing values for the number of centres and subjects. As these could not be included in the multiverse analysis, they were excluded. For the comparison of the estimated amount in the letter of intent, the monocentric group, which had no funding requests exceeding 1 million, was merged with the group comprising 2 to 10 centres. No other changes to the initial protocol occurred during the study.

To evaluate whether the non-significant gender coefficient in the multivariable model may reflect insufficient statistical power, as suggested by a reviewer, we performed a Minimum Detectable Effect (MDE) analysis.

Following a reviewer’s suggestion during peer review, we added an exploratory logistic regression including an interaction term between gender and academic rank to assess whether the association between gender and funding success varied across academic status categories.

## Results

In 2022, 325 funding applications were submitted to PHRC-N. Four studies without participant inclusion were excluded (two retrospective studies and two meta-analyses), leaving 321 letters of intent with 83 (26%) by women and 238 (74%) by men. The majority of principal investigators (183/321, 57%) were full professors. Male investigators were predominantly full professors, had more prior funding and led more surgical projects than their female counterparts. Those results are detailed in Table 1. A total of 102/321 studies (32%) were funded, with no significant gender difference in success rates (77/238 (32%) for men versus 25/83 (34%) for women, OR = 1.11 [95%CI 0.65–1.93], *p* = 0.707). Similarly, preselection rates (126/238 (53%) for men versus 40/83 (48%) for women, OR = 1.21 [95%CI 0.73–2.00], *p* = 0.456) and funding amounts awarded (€781,000 for men versus €715,000 for women, Beta = -65 866.39 [–231 930.04; 100 197.25], *p* = 0.433) showed no gender disparities. The findings remained consistent in multivariate analyses, for success rate (OR = 1,28 [95%CI 0.71–2.34], *p* = 0.412) preselection rates (OR = 1.16 [95%CI 0.67–2.02], *p* = 0.593) and funding (Beta = –42 330.81 [–148 446.97; 63 785.34], *p* = 0.43).

**Table 1:** Description of PHRC-N applications

	All	Men	Women	*p*-value
	321	238 (74%)	83 (26%)	
Preselection				
Accepted	166 (52%)	126 (53%)	40 (48%)	0.46
Funding success				
Accepted	102 (32%)	77 (32%)	25 (30%)	0.71
Academic rank				
Full Professor	183 (58%)	151 (64%)	32 (40%)	** < 0.001**
Associate professor	38 (12%)	25 (11%)	13 (16%)	
Other	96 (30%)	60 (25%)	36 (44%)	
Missing	4	2	2	
Localization				
Ile-de-france	121 (38%)	84 (35%)	37 (45%)	0.13
Province	200 (62%)	154 (65%)	46 (55%)	
Prior funding				
Yes	160 (50%)	131 (55%)	29 (35%)	**0.002**
Discipline				
Surgery	50 (16%)	43 (18%)	7 (8%)	**0.04**
Medicine	271 (84%)	195 (82%)	76 (92%)	
Number of centre				
Monocentric	7 (2%)	5 (2%)	2 (2%)	0.31
[2–10]	114 (36%)	78 (33%)	36 (43%)	
[11–25]	129 (40%)	102 (43%)	27 (33%)	
[26–50]	63 (20%)	48 (20%)	15 (18%)	
[> 50]	8 (2%)	5 (2%)	3 (4%)	
Resubmission				
Yes	95 (30%)	74 (31%)	21 (25%)	0.32
Sample size				
[< 100]	52 (16%)	33 (14%)	19 (23%)	0.1
[100–499]	182 (57%)	135 (57%)	47 (57%)	
[500–1000]	48 (15%)	41 (17%)	7 (8%)	
[> 1000]	39 (12%)	29 (12%)	10 (12%)	
Project budget				
[< 499 k]	89 (28%)	68 (29%)	21 (25%)	0.22
[500-999 k]	177 (55%)	125 (53%)	52 (63%)	
[> 1 M]	55 (17%)	45 (19%)	10 (12%)	

Multivariate analyses are detailed in Table 2. It revealed higher success rates for projects led by investigators based in Ile-de-France (OR = 1.79 [95%CI 1.07–3.01], *p* = 0.027) and for large-scale studies enrolling over 1000 participants (OR = 3.83 [95%CI 1.32–11.87], *p* = 0.016). For the MDE analysis, using the observed standard error of the gender coefficient (SE = 0.302), the study had 80% power at *α* = 0.05. Based on this parameter, we determined that the minimum detectable gender effect corresponded to an odds ratio of approximately 2.33 or larger. This indicates that only large gender differences could have been reliably detected; smaller effects below an OR of 2.3 remain compatible with our data and cannot be excluded.

**Table 2:** Success rate between submission of a letter of intent and funding authorization for PHRC 2023

Variable		Funded (105)	Unfunded (220)	UV OR	UV *p*-value	MV OR	MV *p*-value
Gender							
Men		77 (32%)	161 (68%)				
Women		25 (30%)	58 (70%)	1.11 [0.65;1.93]	0.707	1.28 [0.71;2.34]	0.415
Discipline							
Surgery		10 (20%)	40 (80%)				
Medicine		92 (34%)	179 (66%)	0.49 [0.22;0.98]	0.055	0.55 [0.24;1.18]	0.142
Localization							
Ile-de-france / Paris		50 (41%)	71 (59%)				
Province		52 (26%)	148 (74%)	2 [1.24;3.25]	**0.005**	1.79 [1.07;3.01]	**0.027**
Prior funding							
Yes		53 (33%)	107 (67%)				
No		49 (30%)	112 (70%)	1.13 [0.71;1.81]	0.605	0.98 [0.56;1.69]	0.93
Academic rank							
Full professor		58 (32%)	125 (68%)				
Associate professor		14 (37%)	24 (63%)	0.8 [0.39;1.68]	0.538	0.86 [0.39;1.96]	0.721
Other		30 (31%)	66 (69%)	1.02 [0.6;1.75]	0.94	1.06 [0.58;1.97]	0.85
Number of centres							
Monocentric		1 (14%)	6 (86%)	2.9 [0.47;55.63]	0.332	4.03 [0.57;82.06]	0.226
[11–25]		42 (33%)	87 (67%)				
[2–10]		34 (30%)	80 (70%)	1.14 [0.66;1.97]	0.647	1.13 [0.62;2.07]	0.688
[26–50]		22 (35%)	41 (65%)	0.9 [0.48;1.71]	0.744	0.92 [0.47;1.84]	0.82
[> 50]		3 (38%)	5 (62%)	0.8 [0.19;4.07]	0.773	1.01 [0.2;5.99]	0.987
Sample size							
[< 100]		21 (40%)	31 (60%)				
[100–499]		58 (32%)	124 (68%)	1.45 [0.76;2.72]	0.253	1.73 [0.85;3.53]	0.131
[500–1000]		15 (31%)	33 (69%)	1.49 [0.66;3.44]	0.343	2.19 [0.86;5.72]	0.103
[> 1000]		8 (21%)	31 (79%)	2.62 [1.04;7.14]	**0.047**	3.83 [1.32;11.87]	**0.016**
Project budget							
[< 499 k]		21 (24%)	68 (76%)				
[500-999 k]		64 (36%)	113 (64%)	0.55 [0.3;0.96]	**0.04**	0.54 [0.28;1]	0.056
[> 1 M]		17 (31%)	38 (69%)	0.69 [0.33;1.48]	0.334	0.5 [0.21;1.18]	0.113

*UV* Univariate, *MV* multivariate, *OR* odds ratio

Multivariate analyses exploring the transition from letter of intent to project stage (Supplement 1), from project to financing authorization (Supplement 2) and the comparison of estimated (Supplement 3) and authorized (Supplement 4) funding amounts, and an exploratory model including Gender × Academic Rank interactions (Supplement 5) are provided in the supplementary materials. In the exploratory model including Gender × Academic Rank interactions, the main effect of gender remained non-significant, and most interaction terms showed no evidence of differential effects of academic rank by gender. One interaction (gender × “other” rank) appeared significant but was imprecise and unstable, consistent with a chance finding in a small sample rather than a robust gender-specific effect.

## Discussion

Our study did not identify evidence for a difference between men and women in terms of preselection or securing PHRC-N funding for the year 2022. Furthermore, we found no evidence for a difference in the amount of funding obtained by men and women. These results contrast with previous literature which highlights a gender bias in obtaining funding and in the amounts received in the world [17, 18]. Other studies, particularly in Europe, observe a lower number of funding applications among women [19–21]. However, our results do not imply that men and women have had equal opportunities, or that there are no biases in other aspects of the process, such as submission or thematic choices. Indeed, only a quarter of principal investigators who submitted a proposal were women, despite the increasing feminization of medical research and medicine in France [3, 22]. De facto, there is a gender difference, favouring men, in submitting projects to the PHRC-N. In addition, more male principal investigators were full professors (64 versus 40%) and had received prior research funding by the PHRC compared with their female counterparts (55% versus 35%). Many studies have shown that women face greater challenges in accessing university and high-ranking positions due to discrimination and a lack of mentorship opportunities in medicine. Only 20% of full professor positions are held by women [3, 7]. This limited access to academic hospital positions may restrict the ability to conduct and publish studies, ultimately limiting publication [23–25] and funding applications [26, 27]. Moreover, the PHRC is a highly selective and exclusive call for projects. This privileged nature favours researchers in senior positions, often men, due to the underrepresentation of women in the higher ranks of the academic hierarchy. Clinical studies remain collaborative work involving dozens of people. However, projects are typically led by a single principal investigator, usually the person who leads the research team, most often a man. Women, being less represented in top positions, have fewer opportunities to submit ambitious projects under their own name. Once more, PHRC-N remains highly elitist. So, conclusions of our study cannot be generalized to calls for proposals of smaller scale or those with less funding.

Alternatively, one may argue that our findings may indicate changes in institutional policies and practices aimed at promoting gender equity in medical research, similar to Athena SWAN [28]. Athena SWAN is a framework used worldwide to support and enhance gender equality within higher education and research. To our knowledge, there is no existing policy for the PHRC regarding these aspects, and no such quotas are specified by the PHRC jury. Our findings may simply indicate no bias in the decision process at the funding stage for the French PHRC, contrary to our prior hypothesis.

Among the covariates, used for adjustments, we found that certain factors were associated with securing PHRC funding, such as having a principal investigator based in Paris and conducting research with more than 1000 participants. Beyond the quality of research proposals, the association with the Parisian origin of principal investigators identified in this study may be attributable to multiple factors not examined here, including their experience, affiliation with established research networks, reputation or research productivity. These factors are frequently considered indicators of an institution’s research quality. Furthermore, the reputation of Parisian hospitals, combined with easier patient recruitment due to population density and the presence of many reference centres, might also facilitate access to funding. The advantage of Parisian centres, which benefit from higher population density and better-established research infrastructure, creates a form of geographical bias to the detriment of researchers from other, less densely populated regions. This trend exacerbates a hierarchy in research that can favour institutions already in a strong position, creating a vicious cycle where the academic elite continues to attract funding, without allowing for genuine renewal of teams and research perspectives. Studies with over 1000 subjects often involve rare endpoints, such as mortality, which enhances their clinical relevance. These studies can have a significant impact on clinical practice, which justifies their priority in PHRC funding decisions. The statistical power of these studies is also an important criterion in the selection of funded studies. This reflects the funding priorities of the PHRC-N, which favours large-scale projects. Funding for more targeted but potentially innovative research is not prohibited, but is preferentially allocated to other calls for proposals (e.g., PHRC-I).

Of course, our results must be interpreted cautiously. First, despite our efforts to manage confounding factors with multivariate analyses, residual confounding remains possible in such an observational and retrospective study. For instance, anonymization prevented complete access to proposals, limiting our ability to evaluate their intrinsic quality, which is crucial for selection. Our analysis could be enhanced with additional data, such as bibliometric indices, access to research resources and network sizes, which were also unavailable due to anonymization. Such information could also provide a more detailed understanding of the characteristics of funded studies. In addition, our study did not explore other factors of inequality, such as ethnicity or religion. Ethnic statistics are generally prohibited in France, and the use of a computerized letter of intent for selection helps reduce the influence of such factors on study selection.

Then, our study focuses exclusively on the year 2022 and the PHRC-N, which is a major funding source for clinical research but not the only one. This a call for proposals is mainly sought by experienced principal investigators, often full professors, with several studies to their credit. This limits the scope and generalizability of our findings. Moreover, the wide confidence interval for the gender odds ratio [0.71–2.34] and the low events-per-variable ratio suggest limited statistical power, increasing the risk of Type II error; thus, our inability to detect a significant gender effect (*p* = 0.415) should not be interpreted as evidence of its absence. Furthermore, our MDE analysis, based on the observed standard error of the gender coefficient (SE = 0.302), indicated that only large gender effects (odds ratio > 2.33) could be reliably detected with 80% power at *α* = 0.05. Smaller effects, which remain compatible with our data, could not be excluded.

Lastly, – despite being exhaustive of all research proposals submitted to PHRC-N for the year 2022 – our sample size remains limited, and our study also has a potential lack of power. Subtle differences may exist that we were unable to statistically detect. Therefore, an analysis of data over several years, including other funding opportunities from other programs such as PHRC-I, the Medical-Economic Research Program (PRME), the Health System Performance Research Program (PREPS), or the Translational Research Program (PRT), would provide a more representative perspective. This would allow the detection of a smaller effect size if it exits.

## Conclusions

This study found no evidence of gender disparity in PHRC-N funding outcomes for 2022. However, there was an imbalance between male and female principal investigators submitting an application. Future research should examine long-term trends and broader funding programs to evaluate potential disparities in research opportunities and outcomes.

## Supplementary Information

Supplementary Material 1.

## Data Availability

Anonymised data and code allowing for reproducing the analyses are shared on the Open Science Framework (10.17605/OSF.IO/3CR67).
