# Supplementary material for: Gender disparities in securing national clinical research program (PHRC-N) funding in France: a retrospective analysis of the 2022 campaign
Source: Health Res Policy Syst. 2026 Mar 8;24:35. doi: 10.1186/s12961-026-01450-z (PMC13081353; doi:10.1186/s12961-026-01450-z)
Supplement: Supplementary file 1 — Supplementary Material 1. [file 12961_2026_1450_MOESM1_ESM.docx]

**Supplement1.** Success rate on complete protocol: Moving from letter of intent to project stage

| **Variable** | | **Funded (105)** | **Unfunded (220)** | **UV OR** | **UV p-value** | **MV OR** | **MV p-value** |
| --- | --- | --- | --- | --- | --- | --- | --- |
| Gender | |  |  |  |  |  |  |
|  | Men | 126(53%) | 112(47%) |  |  |  |  |
|  | Women | 40(48%) | 43(52%) | 1.21 [0.73;2] | 0.456 | 1.16 [0.67;2.02] | 0.593 |
| Discipline | |  |  |  |  |  |  |
|  | Surgery | 26(52%) | 24(48%) |  |  |  |  |
|  | Medecine | 140(52%) | 131(48%) | 1.01 [0.55;1.86] | 0.965 | 1.14 [0.59;2.2] | 0.697 |
| Localisation | |  |  |  |  |  |  |
|  | Ile-de-france / Paris | 74(61%) | 47(39%) |  |  |  |  |
|  | province | 92(46%) | 108(54%) | 1.85 [1.17;2.94] | **0.009** | 1.77 [1.08;2.91] | **0.025** |
| Prior funding | |  |  |  |  |  |  |
|  | Yes | 88(55%) | 72(45%) |  |  |  |  |
|  | Non | 78(48%) | 83(52%) | 1.3 [0.84;2.02] | 0.24 | 1.02 [0.62;1.7] | 0.925 |
| Academic rank | |  |  |  |  |  |  |
|  | Full professor | 101(55%) | 82(45%) |  |  |  |  |
|  | Associate professor | 21(55%) | 17(45%) | 1 [0.49;2.01] | 0.994 | 0.95 [0.44;2.04] | 0.899 |
|  | Other | 44(46%) | 52(54%) | 1.46 [0.89;2.4] | 0.138 | 1.54 [0.88;2.73] | 0.136 |
| Number of centers | |  |  |  |  |  |  |
|  | monocentric | 4(57%) | 3(43%) | 0.81 [0.15;3.82] | 0.789 | 1.41 [0.24;7.59] | 0.686 |
|  | [2-10] | 53(46%) | 61(54%) | 1.24 [0.75;2.06] | 0.397 | 1.42 [0.82;2.5] | 0.215 |
|  | [11-25] | 67(52%) | 62(48%) |  |  |  |  |
|  | [26-50] | 37(59%) | 26(41%) | 0.76 [0.41;1.39] | 0.376 | 0.74 [0.38;1.43] | 0.376 |
|  | [>50] | 5(62%) | 3(38%) | 0.65 [0.13;2.75] | 0.564 | 0.73 [0.13;3.48] | 0.699 |
| Sample size | |  |  |  |  |  |  |
|  | [<100] | 33(63%) | 19(37%) |  |  |  |  |
|  | [100-499] | 90(49%) | 92(51%) | 1.78 [0.95;3.4] | 0.076 | 1.85 [0.92;3.83] | 0.09 |
|  | [500-1000] | 27(56%) | 21(44%) | 1.35 [0.61;3.03] | 0.463 | 1.76 [0.71;4.43] | 0.225 |
|  | [>1000] | 16(41%) | 23(59%) | 2.5 [1.08;5.95] | **0.035** | 2.89 [1.08;7.95] | **0.036** |
| Project budget | |  |  |  |  |  |  |
|  | [<499k] | 43(48%) | 46(52%) |  |  |  |  |
|  | [500-999k] | 97(55%) | 80(45%) | 0.77 [0.46;1.28] | 0.318 | 0.87 [0.49;1.51] | 0.611 |
|  | [>1M] | 26(47%) | 29(53%) | 1.04 [0.53;2.05] | 0.903 | 1 [0.46;2.16] | 0.997 |

UV: Univariate. MV: multivariate. OR: odds ratio

**Supplement2.** Success rate from project to financing authorization

| **Variable** | | **Funded (105)** | **Unfunded (220)** | **UV OR** | **UV p-value** | **MV OR** | **MV p-value** |
| --- | --- | --- | --- | --- | --- | --- | --- |
| Gender | |  |  |  |  |  |  |
|  | Men | 77(61%) | 49(39%) |  |  |  |  |
|  | Women | 25(62%) | 15(38%) | 0.94 [0.45;1.95] | 0.875 | 1.29 [0.55;2.98] | 0.552 |
| Discipline | |  |  |  |  |  |  |
|  | Surgery | 10(38%) | 16(62%) |  |  |  |  |
|  | Medecine | 92(66%) | 48(34%) | 0.33 [0.13;0.76] | **0.011** | 0.4 [0.15;1.05] | 0.063 |
| Localisation | |  |  |  |  |  |  |
|  | Ile-de-france / Paris | 50(68%) | 24(32%) |  |  |  |  |
|  | province | 52(57%) | 40(43%) | 1.6 [0.85;3.06] | 0.147 | 1.36 [0.67;2.78] | 0.39 |
| Prior funding | |  |  |  |  |  |  |
|  | Yes | 53(60%) | 35(40%) |  |  |  |  |
|  | Non | 49(63%) | 29(37%) | 0.9 [0.48;1.68] | 0.732 | 1 [0.47;2.13] | 0.998 |
| Academic rank | |  |  |  |  |  |  |
|  | Full professor | 58(57%) | 43(43%) |  |  |  |  |
|  | Associate professor | 14(67%) | 7(33%) | 0.67 [0.24;1.77] | 0.435 | 0.79 [0.24;2.38] | 0.682 |
|  | Other | 30(68%) | 14(32%) | 0.63 [0.29;1.31] | 0.225 | 0.56 [0.22;1.35] | 0.204 |
| Number of centers | |  |  |  |  |  |  |
|  | monocentric | 1(25%) | 3(75%) | 5.04 [0.61;104.94] | 0.171 | 5.18 [0.44;131.48] | 0.222 |
|  | [2-10] | 34(64%) | 19(36%) | 0.94 [0.44;1.98] | 0.869 | 0.87 [0.37;2.01] | 0.746 |
|  | [11-25] | 42(63%) | 25(37%) |  |  |  |  |
|  | [26-50] | 22(59%) | 15(41%) | 1.15 [0.5;2.6] | 0.746 | 1.15 [0.46;2.84] | 0.758 |
|  | [>50] | 3(60%) | 2(40%) | 1.12 [0.14;7.21] | 0.905 | 1.26 [0.14;10.16] | 0.826 |
| Sample size | |  |  |  |  |  |  |
|  | [<100] | 21(64%) | 12(36%) |  |  |  |  |
|  | [100-499] | 58(64%) | 32(36%) | 0.97 [0.42;2.26] | 0.934 | 1.36 [0.51;3.84] | 0.551 |
|  | [500-1000] | 15(56%) | 12(44%) | 1.4 [0.49;4] | 0.526 | 2.44 [0.68;9.06] | 0.174 |
|  | [>1000] | 8(50%) | 8(50%) | 1.75 [0.52;5.99] | 0.365 | 2.89 [0.7;12.56] | 0.147 |
| Project budget | |  |  |  |  |  |  |
|  | [<499k] | 21(49%) | 22(51%) |  |  |  |  |
|  | [500-999k] | 64(66%) | 33(34%) | 0.49 [0.24;1.02] | 0.057 | 0.41 [0.18;0.94] | **0.037** |
|  | [>1M] | 17(65%) | 9(35%) | 0.51 [0.18;1.36] | 0.183 | 0.36 [0.11;1.15] | 0.09 |

UV: Univariate. MV: multivariate. OR: odds ratio

**Supplement3.** Comparison of the estimated amount in the letter of intent, (amount [<499] was used as reference)

| **Variable** | | **UV OR** | | **UV p-value** | | **MV OR** | | **MV p-value** | |
| --- | --- | --- | --- | --- | --- | --- | --- | --- | --- |
|  | | [500-999k] | [>1M] | [500-999k] | [>1M] | [500-999k] | [>1M] | [500-999k] | [>1M] |
| Gender | |  |  |  |  |  |  |  |  |
|  | Men |  |  |  |  |  |  |  |  |
|  | Women | 1.35 [0.75;2.42] | 0.72 [0.31;1.67] | 0.319 | 0.444 | 1.58 [0.82;3.03] | 0.92 [0.34;2.48] | 0.169 | 0.874 |
| Discipline | |  |  |  |  |  |  |  |  |
|  | Surgery |  |  |  |  |  |  |  |  |
|  | Medecine | 1.82 [0.93;3.55] | 1.59 [0.65;3.94] | 0.081 | 0.312 | 1.39 [0.66;2.94] | 1.47 [0.51;4.27] | 0.382 | 0.474 |
| Localisation | |  |  |  |  |  |  |  |  |
|  | Ile-de-france / Paris |  |  |  |  |  |  |  |  |
|  | province | 0.66 [0.39;1.12] | 1.49 [0.7;3.15] | 0.125 | 0.298 | 0.62 [0.34;1.12] | 1.36 [0.57;3.25] | 0.114 | 0.487 |
| Prior funding | |  |  |  |  |  |  |  |  |
|  | Yes |  |  |  |  |  |  |  |  |
|  | Non | 1.01 [0.61;1.68] | 0.88 [0.45;1.72] | 0.965 | 0.701 | 1.2 [0.66;2.19] | 1.22 [0.53;2.82] | 0.547 | 0.643 |
| Academic rank | |  |  |  |  |  |  |  |  |
|  | Full professor |  |  |  |  |  |  |  |  |
|  | Associate professor | 0.9 [0.4;2.01] | 0.71 [0.24;2.11] | 0.793 | 0.54 | 1.01 [0.41;2.47] | 1.15 [0.33;4] | 0.979 | 0.827 |
|  | Other | 0.89 [0.51;1.57] | 0.54 [0.24;1.2] | 0.691 | 0.132 | 0.71 [0.36;1.38] | 0.42 [0.16;1.13] | 0.307 | 0.085 |
| Number of centers | |  |  |  |  |  |  |  |  |
|  | [<10] |  |  |  |  |  |  |  |  |
|  | [11-25] | 2.74 [1.53;4.93] | 6.15 [2.6;14.56] | **0.001** | **0** | 2.87 [1.53;5.39] | 5.02 [1.96;12.9] | **0.001** | **0.001** |
|  | [26-50] | 4.06 [1.8;9.16] | 8.24 [2.81;24.18] | **0.001** | **0** | 3.04 [1.26;7.34] | 3.48 [1.04;11.67] | **0.014** | **0.043** |
|  | [>50] | 1.83 [0.32;10.39] | 5.3 [0.67;42.14] | 0.496 | 0.115 | 1.31 [0.21;8.13] | 3.02 [0.31;29.72] | 0.769 | 0.343 |
| Sample size | |  |  |  |  |  |  |  |  |
|  | [<100] |  |  |  |  |  |  |  |  |
|  | [100-499] | 1.67 [0.88;3.2] | 2.74 [0.74;10.12] | 0.119 | 0.131 | 1.59 [0.78;3.26] | 1.89 [0.48;7.47] | 0.202 | 0.363 |
|  | [500-1000] | 4.28 [1.51;12.13] | 16.61 [3.55;77.78] | **0.006** | **0** | 3.25 [1.05;10.04] | 9.33 [1.83;47.5] | **0.041** | **0.007** |
|  | [>1000] | 3.54 [1.03;12.12] | 36.42 [7.24;183.21] | **0.044** | **0** | 2.53 [0.68;9.47] | 18.51 [3.34;102.54] | 0.168 | **0.001** |

UV: Univariate. MV: multivariate. OR: odds ratio

**Supplement4.** Comparison of commitment authorization amounts

| **Variable** | | **Mean (sd)** | **UV β** | **UV p-value** | **MV β** | **MV p-value** |
| --- | --- | --- | --- | --- | --- | --- |
| Gender | |  |  |  |  |  |
|  | Men | 781243.13 (375561.55) |  |  |  |  |
|  | Women | 715376.74 (251307) | -65866.39  [-231930.04;100197.25] | 0.433 | -42330.81  [-148446.97;63785.34] | 0.43 |
| Discipline | |  |  |  |  |  |
|  | Surgery | 567202.6 (139878.53) |  |  |  |  |
|  | Medecine | 788350.84 (360203.71) | 221148.24  [-7760.48;450056.97] | 0.058 | 39374.11  [-112715.01;191463.23] | 0.608 |
| Localisation | |  |  |  |  |  |
|  | Ile-de-france / Paris | 680817.49 (232798.27) |  |  |  |  |
|  | province | 844087.8 (418526.79) | 163270.31 [25866.11;300674.51] | **0.02** | 63484.81  [-27685.75;154655.37] | 0.17 |
| Prior funding | |  |  |  |  |  |
|  | Yes | 797566.5 (402964.1) |  |  |  |  |
|  | Non | 729857.43 (279282.25) | -67709.07  [-208519.59;73101.46] | 0.342 | -46941.61  [-145017.41;51134.19] | 0.344 |
| Academic rank | |  |  |  |  |  |
|  | Full professor | 767573.2 (377914.49) |  |  |  |  |
|  | Associate professor | 788140.07 (264152.72) | 20566.87  [-189295.49;230429.24] | 0.846 | -25276.14  [-150775.57;100223.29] | 0.69 |
|  | Other | 751030 (340358.51) | -16543.2  [-179101.89;146015.5] | 0.84 | 18277.01  [-89137.43;125691.46] | 0.736 |
| Number of centers | |  |  |  |  |  |
|  | monocentric | 532836 (NA) | -233835.05  [-905089.23;437419.13] | 0.491 | -6135.86  [-452843.78;440572.06] | 0.978 |
|  | [2-10] | 637063.76 (278352.67) | -129607.29  [-285526.47;26311.88] | 0.102 | -37476.24  [-136921.08;61968.59] | 0.456 |
|  | [11-25] | 766671.05 (334533.56) |  |  |  |  |
|  | [26-50] | 925232.29 (386733.27) | 158561.24  [-20108.23;337230.7] | 0.081 | 107611.55  [-13103.93;228327.02] | 0.08 |
|  | [>50] | 1131413.33 (515742.56) | 364742.28  [-32146.36;761630.93] | 0.071 | 207682.65  [-48557.01;463922.3] | 0.111 |
| Sample size | |  |  |  |  |  |
|  | [<100] | 544767.1 (302842.54) |  |  |  |  |
|  | [100-499] | 728484.02 (224859.08) | 183716.92 [28283.24;339150.6] | **0.021** | 58301.85  [-63926.36;180530.06] | 0.345 |
|  | [500-1000] | 1053786.21 (457259.89) | 509019.12 [299950.1;718088.14] | **0** | 113746.73  [-52911.68;280405.14] | 0.178 |
|  | [>1000] | 1098395.38 (458550.32) | 553628.28 [301875.84;805380.72] | **0** | 86146.93  [-115255.93;287549.79] | 0.397 |
| Project budget | |  |  |  |  |  |
|  | [<499k] | 430000.52 (115646.88) |  |  |  |  |
|  | [500-999k] | 732691.62 (194122.6) | 302691.1 [195495.85;409886.35] | **0** | 279079.87 [164260.13;393899.6] | **0** |
|  | [>1M] | 1332668.75 (346258.43) | 902668.23 [762071.76;1043264.69] | **0** | 809314.38 [651253.83;967374.93] | **0** |

UV: Univariate. MV: multivariate. OR: odds ratio

**Supplement5**. the exploratory model including Gender × Academic Rank interactions on success rate between submission of a letter of intent and funding authorization for PHRC 2023

| **Variable** | | | **MV OR** | **MV p-value** |
| --- | --- | --- | --- | --- |
| Gender | | |  |  |
|  | | Men |  |  |
|  |  | Women | 0.65 [0.28;1.5] | 0.306 |
| Discipline | | |  |  |
|  | | Surgery |  |  |
|  |  | Medecine | 0.57 [0.25;1.23] | 0.171 |
| Localisation | | |  |  |
|  | | Ile-de-france / Paris |  |  |
|  |  | province | 1.85 [1.1;3.14] | 0.021 |
| Prior funding | | |  |  |
|  | | Yes |  |  |
|  |  | Non | 1 [0.47;2.13] | 0.998 |
| Academic rank | | |  |  |
|  | | Full professor |  |  |
|  |  | Associate professor | 0.73 [0.29;1.89] | 0.507 |
|  |  | Other | 0.69 [0.36;1.35] | 0.274 |
| Number of centers | | |  |  |
|  | | monocentric | 5.16 [0.7;107.6] | 0.162 |
|  | | [2-10] | 1.05 [0.58;1.94] | 0.746 |
|  | | [11-25] |  |  |
|  | | [26-50] | 0.89 [0.45;1.8] | 0.746 |
|  | | [>50] | 0.88 [0.17;5.35] | 0.878 |
| Sample size | | |  |  |
|  | | [<100] |  |  |
|  |  | [100-499] | 1.63 [0.79;3.36] | 0.181 |
|  |  | [500-1000] | 2.08 [0.8;5.49] | 0.135 |
|  |  | [>1000] | 3.55 [1.21;11.12] | **0.024** |
| Project budget | | |  |  |
|  | | [<499k] |  |  |
|  |  | [500-999k] | 0.54 [0.28;1.01] | 0.059 |
|  |  | [>1M] | 0.52 [0.22;1.24] | 0.141 |
| Interaction term | | |  |  |
|  | Women x associate professor | | 2.4 [0.41;16.17] | 0.342 |
|  | Women x other academic rank | | 4.48 [1.25;16.71] | **0.023** |

UV: Univariate. MV: multivariate. OR: odds ratio
